# Modulation of Kex2p Cleavage Site for In Vitro Processing of Recombinant Proteins Produced by *Saccharomyces cerevisiae*

**DOI:** 10.4014/jmb.2306.06024

**Published:** 2023-07-07

**Authors:** Mi-Jin Kim, Se-Lin Park, Seung Hwa Kim, Hyun-Joo Park, Bong Hyun Sung, Jung-Hoon Sohn, Jung-Hoon Bae

**Affiliations:** 1 Synthetic Biology Research Center, Korea Research Institute of Bioscience and Biotechnology, Daejeon 34141, Republic of Korea; 2 Department of Food Science and Technology, Chungnam National University, Daejeon 34134, Republic of Korea; 3 Department of Biosystem and Bioengineering, KRIBB School of Bioscience, Korea University of Science and Technology (UST), Daejeon 34113, Republic of Korea; 4 Cellapy Bio Inc., Bio-Venture Center 211, Daejeon 34141, Republic of Korea

**Keywords:** Recombinant protein, secretion, Kex2p, in-vitro processing, *Saccharomyces cerevisiae*

## Abstract

Kex2 protease (Kex2p) is a membrane-bound serine protease responsible for the proteolytic maturation of various secretory proteins by cleaving after dibasic residues in the late Golgi network. In this study, we present an application of Kex2p as an alternative endoprotease for the in vitro processing of recombinant fusion proteins produced by the yeast *Saccharomyces cerevisiae*. The proteins were expressed with a fusion partner connected by a Kex2p cleavage sequence for enhanced expression and easy purification. To avoid in vivo processing of fusion proteins by Kex2p during secretion and to guarantee efficient removal of the fusion partners by in vitro Kex2p processing, P_1_', P_2_', P_4_, and P_3_ sites of Kex2p cleavage sites were elaborately manipulated. The general use of Kex2p in recombinant protein production was confirmed using several recombinant proteins.

## Introduction

Yeast is a popular eukaryotic host for recombinant protein production because it can be easily cultured to high cell densities and efficiently secrete proteins after post-translational modification similar to those in higher eukaryotic cells [1, 2]. Secretory production of recombinant proteins fused with fusion partners and in vitro processing with sequence specific endo-proteases is favored because it greatly simplifies the purification procedure compared with intracellular production. For this purpose, Factor Xa protease and enterokinase (EK) are generally used because they can produce intact target proteins when fusion partners are fused to the N-terminus of target proteins. However, they frequently cleave at other nonspecific basic residues [3-5] and cost of these proteases is too high to use in large-scale production of recombinant proteins.

The product of *Saccharomyces cerevisiae* KEX2 (Kex2p, E.C. 3.4.21.61) is a Ca^2+^-dependent membrane-bound serine protease [6, 7]. Kex2p matures K1 killer toxin [8] and mating factor a (MFa) [9] prohormones by cleaving after dibasic residues (Lys-Arg and Arg-Arg) in the late Golgi network. Because dibasic residues are frequently found in proteins, exceptionally high substrate specificity is required for Kex2p to discriminate non-substrate proteins [10]. These characteristics imply that substrate specificity of Kex2p can be modulated by changing the amino acids adjacent to the cleavage site [11-13]. In addition, Kex2p is highly efficient because this membrane-bound enzyme is shortly exposed to correct substrate proteins mixed with a lot of non-substrate proteins in the trans-Golgi network. This process is very similar to the processing of mammalian pro-proteins by mammalian homologues of Kex2p [for example, furin and pro-protein convertase family (PCs)], implying conservation of this process throughout eukaryotic evolution [14]. Kex2p cleaves several mammalian pro-proteins, such as human pro-albumin [15], protein C precursor [16], and insulin-like growth factor-1[17].

Many studies have been conducted to exploit these advantages for the production of recombinant proteins. When the amount of recombinant proteins exceeds processing capacity of Kex2p in yeast, overexpression of KEX2 enhances the secretion of recombinant proteins [18]. In addition, modification of the P_1_' site to induce exposed loop structure of Kex2p recognition site led to the enhanced secretion of target proteins in *Pichia pastoris* [19]. Accordingly, soluble Kex2p variants have been produced by truncating the transmembrane (TM) domain in *S. cerevisiae* [20-22] and applied for in vitro processing of fusion proteins [20]. To produce fusion proteins joined by the Kex2p cleavage sequence without in vivo processing in yeast, a *kex2* mutant strain should be used. Although KEX2 is not an essential gene, the null mutant is very sensitive to various stresses, and growth in complex media is severely retarded. Therefore, the *kex2* mutant strain was not an appropriate host for recombinant protein expression. When other expression systems are used to express recombinant proteins, such as bacteria and cell lines, Kex2p can be used without considering the *kex2* mutant.

In this study, instead of using *kex2* mutant strain, Kex2p cleavage sites of fusion proteins were elaborately manipulated using the ProP 1.0 server, which predicts cleavage sites for furin and PCs to enhance or repress in vivo processing in yeast and to guarantee in vitro processing with Kex2p.

## Materials and Methods

### Strains, Chemicals, Media, and Analysis of Secreted Proteins

*Escherichia coli* DH5a [F− *lacZΔ* M15 *hsd*R17(r-m-) *gyr*A36] was used for the plasmid constructions. *S. cerevisiae* allgal (Mat α *pep4*::*HIS3 prb1*, *can1*, *his3-200*, *gal80*, *gal1*, *gal2*, *gal7*, *gal10*) [23] was used as a general host for recombinant protein expression. Q5 High-Fidelity DNA Polymerase and restriction endonucleases were purchased from New England Biolabs (USA) and used for genetic manipulation. DNA purification was performed using Wizard SV Gel and the PCR Clean-Up System (Promega, USA). Yeast transformation was performed using the lithium acetate method [24]. Yeast transformants were selected by cultivation at 30°C in synthetic defined medium lacking uracil (SD-URA; 0.67% yeast nitrogen base without amino acids, 2% glucose, 0.77 g/l -uracil dropout supplement, and 2% agar). YPD medium (1% yeast extract, 2% peptone, and 2% glucose) was used to express recombinant proteins. Transformants were cultivated in a 10-ml test tube containing 2 ml of YPD broth medium for 40 h with vigorous shaking. Subsequently, 0.6 ml of culture supernatant was precipitated by centrifugation at 10,000 g for 20 min with 0.4 ml of cold acetone and analyzed using SDS-PAGE on 12%polyacrylamide gels (Bio-Rad, USA) under denaturing conditions and then stained with Coomassie blue. To determine the molecular weight of glycoproteins after deglycosylation, precipitated culture supernatants were treated with endoglycosidase H (Promega).

### Plasmid Constructions

**Fig. 1:**
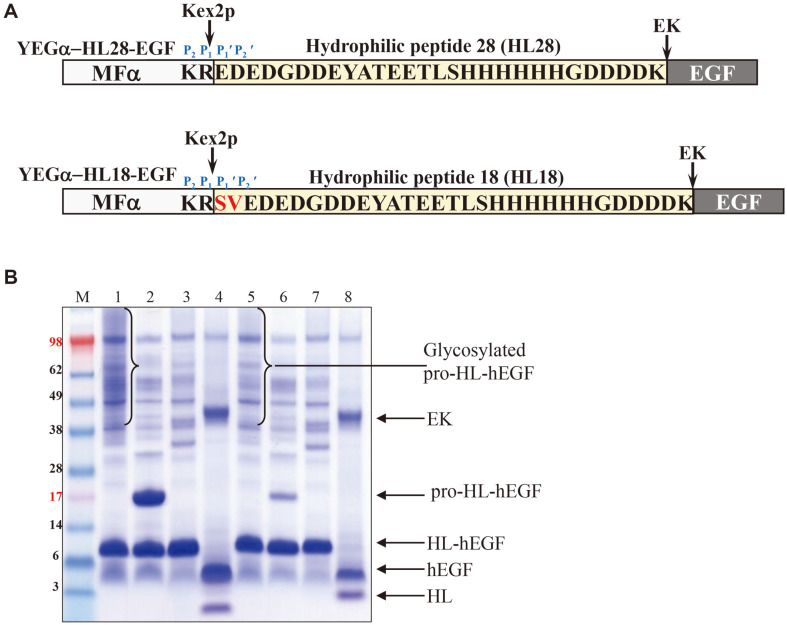
P_1_' and P_2_' site engineering to increase in vivo processing of MFα propeptide. (**A**) Schematic diagram of YEGα-HL28-EGF and YEGα-HL18-EGF vector. The amino acid sequence of HL peptide are indicated. (**B**) SDS-PAGE analysis of the culture supernatant expressing YEGα-HL28-EGF (lanes 1–4) and YEGα-HL18-EGF (lanes 5–8). Concentrated culture supernatants (lanes 1 and 5). Deglycosylated culture supernatants (lanes 2 and 6). Culture supernatants treated with Kex2p (lanes 3 and 7). Culture supernatants treated with EK (lanes 4 and 8). M: protein molecular weight marker.

For the construction of expression vectors, YEGα-HL18-EGF, Ser-Val peptide was added to the N-terminus of the HL domain of the YEGα-HL28-EGF vector that secretes human EGF fused with the HL tag by the MFα signal-peptide under the control of *GAL10* promoter [25] by using Polymerase Chain Reaction (PCR) (Fig. 1). The MFα signal-peptide gene containing the linker peptide (underlined) (MRFPSIFTAVLFAASSALAAPVNTTTEDET AQIPAEAVIGYL DLEGDFDVAVLPFSNSTNNGLLFINTTIASIAAKEEGVAASASAGLALDKR) and HL28-hEGF gene connected by six histidine codon and enterokinase cleavage site (underlined) (EDEDGDDEYATEETL SHHHHHHGDDDDKNSDSECPLSHDGYCLHDGVCMYIEALDKYACNCVVGYIGERCQYRDLKWWELR) were amplified from the YEGα-HL28-EGF vector using the primer set (GP-F: 5'-AGTAAGAATTTTTGAAAA TTCAAGGAATTC-3' and MF-R: 5'-ATC-TTCAACAGATCTTTTATCTAAGGCGAG-3') and (MF-F: 5'-AAAAGAT C TGTTGAAG-ATGAAGATGGTGAC-3' and GT-R: 5'-TATATATATTGTCACT CCGTT CA AGTCGAC-3'), respectively. These PCR products were annealed into a single fragment by overlap-extension PCR with GP-F and GT-R primers, and then cloned into the EcoRI/SalI (underlined) site of the YEGα-HL28-EGF vector after digestion with the same restriction endonucleases. To construct YEGα-HK1-EGF, an additional peptide was added to the C-terminus of the HL domain of the YEGα-HL18-EGF vector by overlap extension PCR using primers HK1-R (5'-CACTCGGAGTCGGAGTTTCTCTTAGCTTCACC CTTATCGTCATCGTC-3') and hEGF-F (5'-AACTCCGACTCCGAGTGTCCTC-3'). YEGα-HK2-EGF, YEGα-HK3-EGF, and YEGα-HK4-EGF were constructed using the same method but with the primers HK2-R (5'-CACTCGGAGTCGGAGTTT CTCTTAGCAGCACCCTTATCGTCATCGTC-3'), HK3-R (5'-CACTCGGAGTCGGAGTTTCTCTTCTCAG CACCCTTATCGTCATCGTC-3'), and HK4-R (5'-CACTCGGAGTCGGAGTTTCTCTTCTTTCTACCCTTA TCGTCATCGTC-3'), respectively, instead of HK1-R primer.

To construct YGaT10-HK2-*CbPHY*, the phytase gene from *Citrobacter braakii* (*CbPHY*, AY471611.1) fused with the HK2 tag was synthesized based on the codon usage of *S. cerevisiae* and then cloned into the YGaTFP10 vector [26] using in vivo recombination. Exendin-4 gene from *Heloderma suspectum* (*EXD4*, U77613.1) and defensin-2 from *Zea mays* (*ZmBD2*, NM001153491.2) fused with the HK2 tag (HK2-*EXD4* and HK2-*ZmBD2*) were synthesized and cloned into the YGaTFP1 vector [26] to construct YEGα-HK2-*EXD4* and YEGα-HK2-*ZmBD2*. The HK2 tag of these vectors was modified into HK1, 3, 5, 6, and 7(Fig. 4D) using overlap extension PCR.

### Fermentation and Protein Purification

For the fermentation of recombinant strains, seed culture was prepared using 200 ml of SD-URA broth contained in a 1,000-ml Erlenmeyer flask, and incubated overnight at 30°C. The cultured seed (200 ml) was inoculated into a 5-L jar fermentor (Kobiotech, Korea) containing 1.8 L of medium consisting of 2% glucose, 40 g yeast extract, and 10 g peptone (per liter). After glucose consumption, a feeding medium containing 300 g glucose, 300 g galactose, and 150 g yeast extract (per liter) was added. The hourly feeding rate was manually increased from 2 to 10 g/l according to cell growth. The pH (5.5) was controlled using NH3 (25%). For the analysis of secreted proteins from fed-batch fermentation, 10 ml of culture supernatant was directly used for SDS-PAGE after mixing with 2×SDS-PAGE sample buffer.

HL-hEGF proteins were purified by immobilized metal-affinity chromatography (IMAC) on a nickel-NTA agarose column (Promega) using a low-pressure liquid chromatography (Bio-Rad HR system). Fermentation broth was filtered using 0.1 μm Sartoclear (Sartorius AG, Germany), concentrated five times using 30,000 NMWC Quick-stand (Amersham-Pharmacia Biotech, USA), and adjusted to buffer A [50 mM Tris-HCl (pH 8.0) and 0.5 M NaCl]. After loading onto the column at a flow rate of 1 ml/min, the proteins were eluted in a gradient of buffer B [50 mM Tris-HCl (pH 8.0), 0.5 M NaCl, and 0.5 M imidazole]. The fractions containing the protein of interest were concentrated and exchanged into Kex2p working buffer [50 mM Tris-HCl (pH 8.0), 50 mM NaCl, and 2 mM CaCl_2_] using a 10,000 MWCO Amicon Ultra centrifugal filter device (Millipore, USA). The HL-tag was separated by digestion with CaKex2p [22], which was prepared in-house. One microgram of CaKex2p was used per 1 mg of fusion protein and was incubated at 30°C for 1 h. Intact hEGF was obtained by repeated metal-affinity chromatography on a nickel-NTA agarose column using low-pressure liquid chromatography. The molecular weights of the purified proteins were determined using ProteinWorks (Korea).

### Analysis of hEGF Activity

The bioactivity of hEGF was determined by proliferation assays using HaCaT cells. Cells were cultured with growth media [DMEM (Thermo Fisher, USA) + 10% FBS (Thermo Fisher) + 100 u/ml Penicillin-Streptomycin (Thermo Fisher)] in a humidified incubator containing 5% CO_2_ at 37°C. After cultivation to 80–90% confluence in T75 culture flasks, the medium was replenished with DMEM + 100u/ml Penicillin-Streptomycin supplemented with different concentrations of hEGF and cultured for 48 h before harvesting. After removing the growth media, the cells were incubated for 1 h at 37°C with Ez-Cytox solution (Daeil Lab Service Co Ltd., Korea). Absorbance was measured at 490 nm using a microplate reader (Molecular devices, USA). Statistical comparison of growth was performed using the Student’s *t*-test with a two-tailed distribution (Microsoft Excel). Values were considered statistically significant at *p* value of < 0.05.

## Results

### Secretory Expression of hEGF with HL Fusion Partner in *S. cerevisiae*

When hEGF was expressed with the hydrophilic 28-amino acid peptide (HL28 fusion partner) from Voa1p using the YEGa-HL28-hEGF vector under the control of *GAL10* promoter [25] in *S. cerevisiae*, although the expression of hEGF was greatly improved, a large amount of unprocessed MFa pro-H28L-hEGF fusion protein was detected in the culture broth as smear protein bands due to the hyper-mannosylation with varying extents (Fig. 1B, lane 1). HL28-hEGF does not contain a glycosylation site and the MF pro-peptide contains three glycosylation sites; therefore, secretion of hyper-glycosylated fusion proteins means processing of the MFa pro-peptide by Kex2p in the Golgi network was incomplete. Since the substrate specificity of Kex2p is related to amino acids adjacent to the dibasic cleavage site [11, 12], to increase processing of MFa pro-peptide by Kex2p, the P_1_' and P_2_' positions, i.e. the N-terminus of the HL domain was modified using the ProP 1.0 server (https://services.healthtech.dtu.dk/service.php?ProP-1.0) [27], which predicts pro-peptide cleavage sites in eukaryotic protein sequences using the furin and PCs neural networks. If the ProP score is >0.5, the residue is predicted to be a pro-peptide cleavage site, and a higher ProP score is more confident in the prediction. Although the substrate specificity of furin is slightly different from that of Kex2p, using this program, serine and valine were selected as the P_1_' and P_2_' residues that showed the highest ProP score, regardless of P_3_' and P_4_' residues. After the addition of Ser-Val to the N-terminus of the HL domain, the ProP score increased from 0.220 to 0.567. The expression of the modified vector (YEGa-HL18-hEGF) was compared with that of the original vector (YEGa-HL28-hEGF) by SDS-PAGE of culture supernatants of transformed yeasts. The hyper-glycosylated MFa pro-HL-hEGF fusion proteins expressed from the modified vector were significantly decreased compared to the original vector (compare smear bands of lanes 1 and 5 of Fig. 1B) and this decrease was clearly demonstrated after deglycosylation (compare pro-HL-hEGF bands of lanes 2 and 6 of Fig. 1B) and after treatment with Kex2p (Fig. 1B, lanes 3 and 7), implying increased processing of the MFa pro-peptide by Kex2p in the Golgi complex. The HL fusion partners were easily separated from the fusion proteins by digestion with EK (Fig. 1B, lanes 4 and 8).

### Engineering of Kex2p Cleavage Sites for In Vitro Processing of Fusion Proteins

**Fig. 2:**
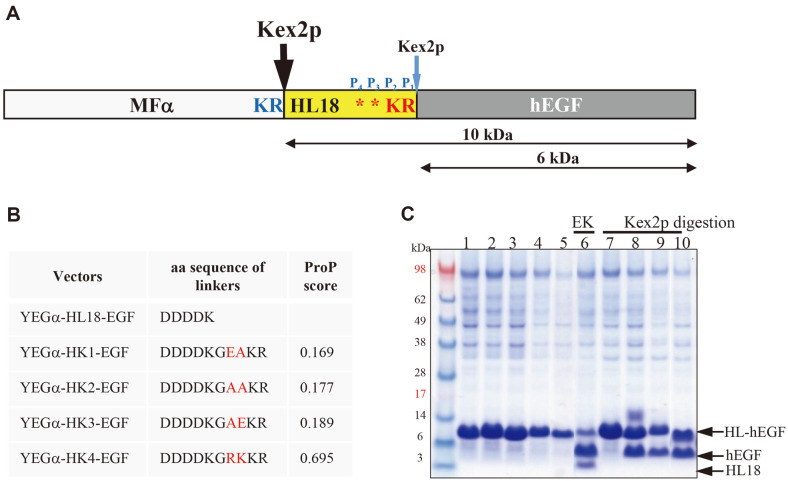
P_3_ and P_4_ site engineering for in vitro processing of HL-hEGF fusion proteins. (**A**) Schematic diagram of the MFα-HL18-hEGF fusion protein. (**B**) Amino acid sequences and ProP scores of modified linkers. (**C**) SDS-PAGE analysis of concentrated culture supernatants of transformants expressing YEGα-HL18-EGF (lane 1), YEGα-HK1-EGF (lane 2), YEGα-HK2-EGF (lane 3), YEGα-HK3-EGF (lane 4), YEGα-HK4-EGF (lane 5), concentrated culture supernatants of transformants expressing YEGα-HL18-EGF after digestion with EK (lane 6), concentrated culture supernatants of transformants expressing YEGα-HK1-EGF (lane 7), YEGα-HK2-EGF (lane 8), YEGα-HK3-EGF (lane 9), and YEGα-HK4-EGF (lane 10) after digestion with Kex2p. M: protein molecular weight marker.

Although EK is a generally used endo-protease for the processing of fusion proteins, its application is restricted in laboratories because of nonspecific cleavage at other basic residues and high cost. First, to use Kex2p instead of EK for the in vitro processing of hEGF fusion proteins, a Kex2p cleavage site was generated by adding Arg just behind the EK cleavage site (DDDD**KR**, ProP 0.162). However, the hEGF fusion protein was not processed despite prolonged incubation with excess Kex2p (data not shown). Therefore, the P_3_ and P_4_ sites, instead of the P_1_' and P_2_'positions of the newly introduced Kex2p cleavage sequence, were manipulated by adding three residues at the P_3_, P_4_ and P_5_ sites based on ProP 1.0 analysis because the modification of the P_1_' and P_2_' sites changed the N-terminus of hEGF. Four kinds of expression vector variables at P_3_ and P_4_ sites were constructed, and the expression of hEGF fusion proteins was compared with that of YEG-HL18-hEGF constructed previously. ProP scores were adjusted to 0.167–0.695 by changing the P_3_ and P_4_ sites (Fig. 2B). The amount of secreted hEGF fusion proteins was slightly affected by the modification of linker sequences, but Kex2p processing was significantly influenced by the P_3_ and P_4_ residues. All variants were secreted as HL-hEGF fusion proteins without in vivo processing by Kex2p (Fig. 2C, lanes 2–5). To confirm in vitro processing of HL-hEGF fusion proteins by Kex2p, Kex2p digested protein bands (Fig. 2C, lanes 7–10) were compared with HL18-hEGF digested with EK (Fig. 2C, lane 6). Unlike other variants, the HK1-hEGF variant (ProP 0.167) was not cleaved by Kex2p, and with the increase in ProP score, HK2-hEGF, HK3-hEGF, and HK4-hEGF were partially processed with Kex2p (compare Fig. 2C, lanes 7 and 8–10). Although the HL tags liberated from HK2-hEGF, HK3-hEGF, and HK4-hEGF by digestion with Kex2p were larger by 5 amino acids than that from the HL18-hEGF digested with EK, they were not identified on SDS-PAGE, contrary to HL fusion partners digested with EK (Fig. 2C, compare lanes 6 and 8–10). Therefore, when the HL-hEGF fusion proteins were digested with Kex2p, the HL fusion partner appeared to be further processed into small peptides. Ultimately, to block the in vivo processing and to allow the in vitro processing of the HL-hEGF fusion protein by Kex2p, the ProP score should be higher than 0.169.

### Production of Intact hEGF Using In Vitro Processing by Kex2p

**Fig. 3:**
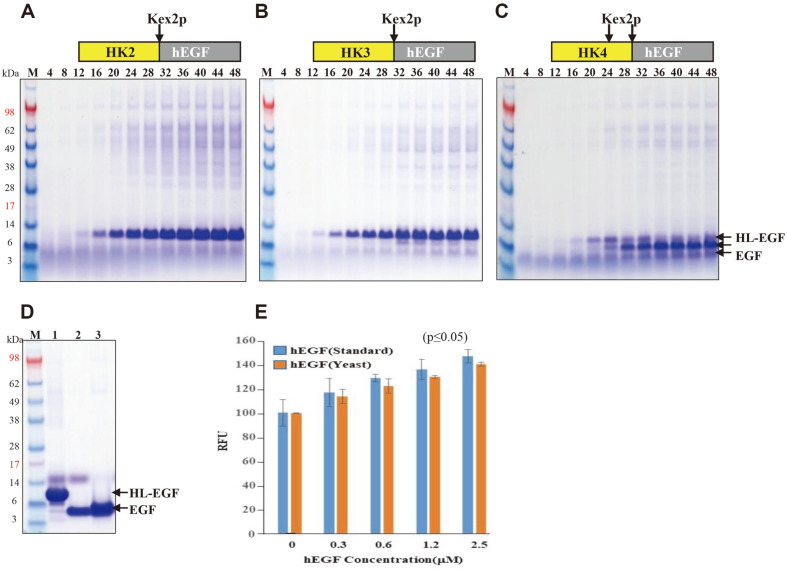
Production of HL-EGF variants by fed-fermentation and IMAC purification. SDS-PAGE analysis of culture broth (10 μl) of strains expressing (**A**) HK2-EGF, (**B**) HK3-EGF, and (**C**) HK4-EGF at the indicated times. Structure of fusion proteins are indicated. HL-EGF and EGF bands are indicated by arrows. (**D**) Purification of HK2-EGF by IMAC. lane 1: eluted fraction from 1^st^ IMAC, lane 2: after digestion with Kex2p, lane 3: unbound fraction from 2^nd^ IMAC. M: protein molecular weight markers. (**E**) Bioactivity assay of the purified hEGF.

To confirm in vivo processing during prolonged cultivation and large-scale production of intact hEGF by in vitro processing with Kex2p, fed-batch fermentations of recombinant *S. cerevisiae* 2805 allgal strains expressing three HL-hEGF variants were conducted. The growth of the three strains reached 120–130 OD_600_ similarly after 48 h of fermentation, but the amount and pattern of secreted HL-hEGF fusion proteins were significantly different. HK2-hEGF was produced as a fusion protein without in vivo processing, similar to the test tube culture (Fig. 3A), but HK3-hEGF was partially processed after 24 h of fermentation and HK4-hEGF was more significantly processed than HK3-hEGF (Figs. 3B and 3C). HK4-hEGF was produced as a mixture of fusion proteins and hEGF. The smaller protein band than that of HK4-hEGF seemed to be a misprocessed fusion protein at the P_3_ position of the linker because the P_4_ and P_3_ sites of HK4-hEGF are dibasic residues (Arg-Lys). The HK2-hEGF fusion protein was purified by IMAC using A poly-histidine tag and then digested with Kex2p to remove the HL fusion partner. HK2-hEGF was completely digested to hEGF and intact hEGF was easily obtained by the 2^nd^ IMAC (Fig. 3D). Purified hEGF demonstrated similar growth-stimulating activities to commercial hEGF (SIGMA) in the growth of HaCaT cells (Fig. 3E). Because the affinity tag is contained in the HL fusion partner, hEGF processed during secretion is not captured by IMAC; therefore, the recovery yield of HK2-hEGF was higher than those of HK3-hEGF and HK4-hEGF at 1^st^ IMAC. Finally, the recovery of intact hEGF from HK2-hEGF was more than two-fold higher than those from HK3-hEGF and HK4-hEGF (Table 1). Therefore, to completely block the in vivo processing of the HL-hEGF fusion protein by Kex2p during fermentation, the ProP score should be lower than that of HK3-hEGF (0.189).

### General Application and Optimization of In Vitro Processing by Kex2p

**Fig. 4:**
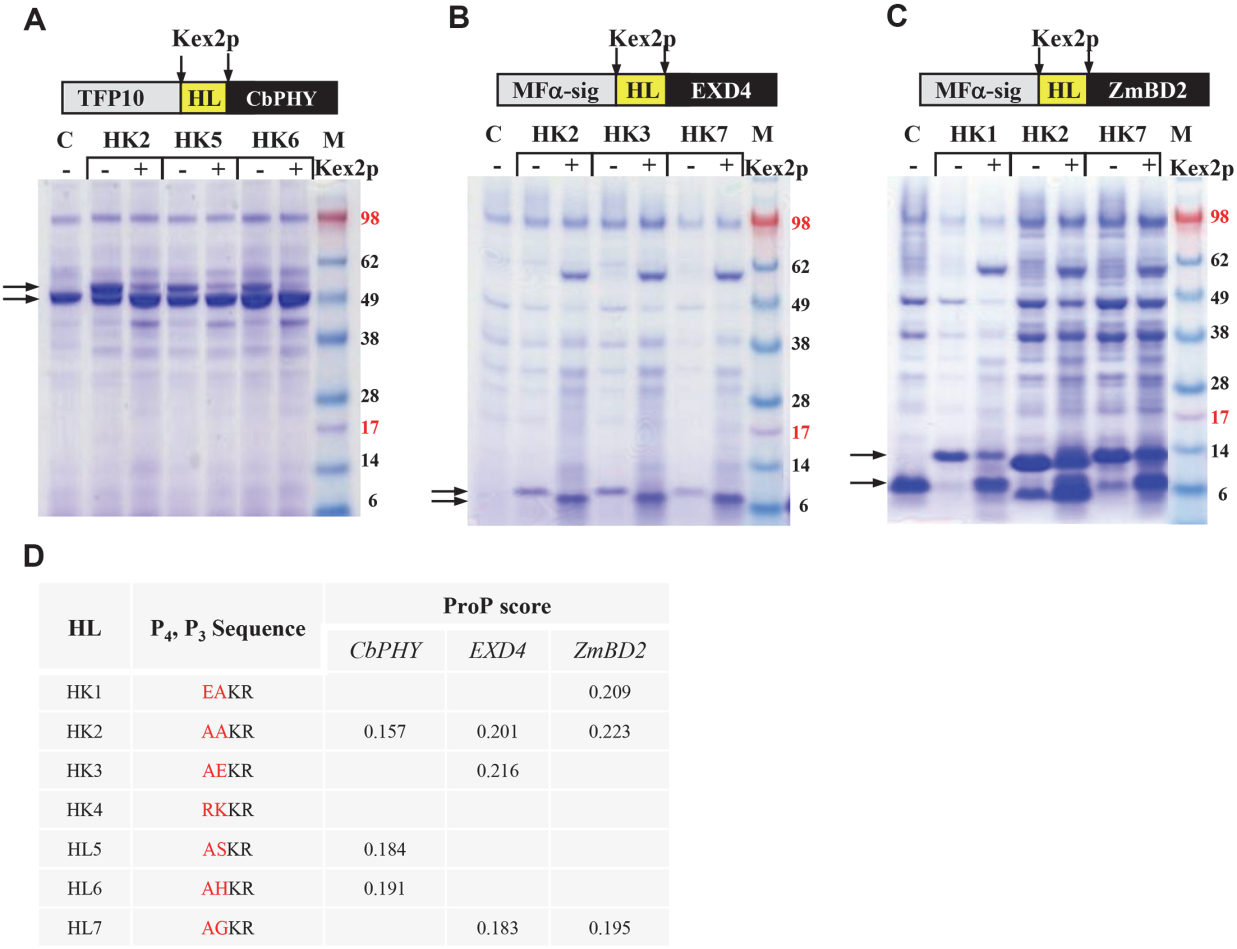
In vitro processing of fusion proteins containing engineered linkers. SDS-PAGE analysis of culture supernatant of (**A**) HL-*CbPhy*, (**B**) HL-*EXD4*, and (**C**) HL-*ZmBD2*, with and without Kex2p digestion. Structure of fusion proteins are indicated. C: proteins expressed without HL fusion partner. The corresponding protein bands are indicated by arrows. M: protein molecular weight markers. (**D**) Amino acid sequence and ProP score of the modified linkers.

To confirm the general application of Kex2p for the in vitro processing of fusion proteins in yeast, phytase, exendin-4, and defensin-2 were expressed with HL fusion partners. Phytase is a phosphatase that catalyzes the hydrolysis of phytic acids found in many plant tissues. Exendin-4 is a potent glucagon-like peptide-1 receptor agonist that promotes insulin secretion, and defensins are host defense peptides (typically 18–45 amino acids long) with antimicrobial activity against bacteria and fungi. Phytase from *Citrobacter braakii* (*CbPHY*) [28], exendin-4 from *Heloderma suspectum* (*EXD4*) [29] and defensin-2 from *Zea mays* (*ZmBD2*) [30] were expressed with HL fusion partners, and the ProP score of Kex2p cleavage sites between HL fusion partner and target proteins was adjusted to 0.157–0.223 (*CbPhy*: 0.159–0.191, *EXD4*: 0.183–0.216, and *ZmBD2*: 0.195–0.223) by modifying the P_3_ and P_4_ sites. The expression of *CbPhy* and *ZmBD2* was slightly enhanced by tagging with HL (Fig. 4A and 4C), whereas *EXD4* was expressed only when HL was fused (Fig. 4B). In the case of *CbPhy*, even though the ProP score of HK2-CbPhy (0.157) was lower than that of HK1-EGF (0.169, Fig. 2B), a significant amount of fusion proteins was in vivo processed by Kex2p. In contrast, the ProP score of HK3-*EXD4* (0.216) was higher than that of HK3-EGF (0.189, Fig. 2B), and in vivo processing did not occur. In case of HL-*ZmBD2*, in vivo Kex2p processing was controlled as expected. HK1-*ZmBD2* and HK7-*ZmBD2* were expressed without in vivo processing, and most of fusion proteins were processed by incubation with Kex2p. These results indicate that Kex2p processing does not correctly correspond to the ProP score according to the target proteins but can be optimized by adjusting the ProP score to approximately 0.15–0.20, by modulation of the P_3_ and P_4_ sites.

## Discussion

For the production of intact recombinant proteins, N-terminal fusion partners and endoproteases cleaving the C-terminus of recognition sites, such as Factor Xa protease, enterokinase, and Kex2p, are usually employed because they remove fusion tags without leaving any unwanted amino acid residues on the target proteins. Although Kex2p is a commercialized endoprotease that cleaves specific sequences, it is not generally used for the production of recombinant proteins compared to other endoproteases because of concerns about substrate specificity, high price, and the requirement of *kex2* mutant strain in yeast.

In this research, to use Kex2p for the production of recombinant protein in yeast without using *kex2* mutant strain, a controlled Kex2p cleavage system was developed by elaborate modification of Kex2p cleavage sites without affecting intactness of target proteins. Since dibasic moieties are frequently found in proteins, Kex2p is highly dependent on the flanking dibasic cleavage sites to discriminate non-substrate sites in vivo. The effects of flanking dibasic cleavage sites on the specificity of Kex2p have been analyzed using synthetic substrates or recombinant proteins [9-12, 31]. Kex2p prefers amino acids with a positive charge to those with a negative charge at the P_4_ and P_3_ sites. Residues with bulky side chains and β-carbon branched side chains were disfavored at the P'1 site. Overall, flanking residues that contribute to the exposed and flexible structure of the cleavage loci are favorable for cleavage by Kex2p. Based on these findings, Ducket *et al*. developed the ProP 1.0 server (https://services.healthtech.dtu.dk/service.php?ProP-1.0), which predicts arginine and lysine pro-peptide cleavage sites in eukaryotic protein sequences using an ensemble of neural networks [27].

When the HL-hEGF fusion protein was expressed using the MFa pro-peptide, despite the C-terminus of the cleavage site (P'1 residue, Glu) is not a reluctant residue for Kex2p, the processing of MFa pro-peptide from HL-hEGF fusion protein by Kex2p was not sufficient. Thus, the N-terminus of the HL domain was modified using ProP 1.0 server. After the addition of 20 amino acids to the N-terminus of the HL fusion partner, the ProP score was compared. When Ser was added, the ProP score was the highest and further increased to 0.567 with the addition of Val at the P'2 site. As expected, in vivo processing of the MFa pro-peptide by Kex2p was significantly improved by the addition of the Ser-Val dipeptide to the N-terminus of the HL fusion partner (Fig. 1). The addition of Ser-Val dipeptide at the P'1 and P'2 sites guaranteed the highest ProP score regardless of the P'_3_ and P'_4_ sites in the ProP score analysis using different proteins. Therefore, the Ser-Val dipeptide sequence can be generally used to improve the processing of fusion proteins by Kex2p in yeast.

Unlike the Kex2p cleavage site after the pro-peptide, for the production of recombinant proteins as fusion proteins with fusion partners, the Kex2p cleavage site between fusion partners and target proteins should be adjustable to block in vivo processing and guarantee in vitro processing. Although the ProP server predicts pro-peptide cleavage sites when the ProP score is >0.5, there are many pro-peptides efficiently cleaved by Kex2p with a ProP score < 0.5, such as MF and K1 killer toxin. To produce four recombinant proteins with fusion partners, the ProP score of the Kex2p cleavage site was adjusted by modifying the P_4_ and P_3_ sites. The optimal ProP score guaranteeing in vitro Kex2p processing without in vivo processing was varied on target proteins but optimized at approximately 0.15–0.20. In some cases, modulation of Kex2p cleavage site resulted in a decrease in total expression levels of the target proteins (HK1, Fig. 4C). Therefore, the amino acids at the P_4_ and P_3_ sites lowering expression of target proteins should be excluded.

When the N-termini of the target proteins are favorable for Kex2p cleavage, the ProP score should be lowered to block in vivo processing by changing the P_4_ or P_3_ sites to reluctant amino acids and vice versa. There were some exceptions to the application of this method for the production of some recombinant proteins. For example, the ProP score of parathyroid hormone (PTH) fused with the HL fusion partner was not lowered to an uncleavable score by changing the P_4_ or P_3_ sites because the N-terminal sequence of PTH is Ser-Val, guaranteeing the highest ProP score. Consequently, it was impossible to produce an HL-PTH fusion protein without in vivo processing. In contrast, in the case of epidermal growth factor from chicken (gEGF), although the ProP score of the HL-gEGF fusion protein increased to <0.5 after modification of the P_4_ or P_3_ sites and the N-terminal residues were favorable for Kex2p cleavage, the fusion tag was not cleaved after incubation with an excess amount of Kex2p.

Considering that the ProP server was constructed based on the furin and PC-specific network, and the substrate specificity of furin and PCs is slightly different from that of Kex2p, the ProP server cannot precisely predict the processing of all pro-proteins by Kex2p. Nevertheless, modulation of the Kex2p recognition site based on the ProP score for the controlled processing of fusion proteins by Kex2p was demonstrated. The system developed in this study may increase the use of Kex2p for the production of recombinant proteins in yeast.

## Figures and Tables

**Table 1:** Purification table for hEGF.

Procedure	HK2-hEGF		HK3-hEGF		HK4-hEGF	
	Total protein (mg)	Yield (%)	Total protein (mg)	Yield (%)	Total protein (mg)	Yield (%)
Fermentation sup	930	100	858	100	650	100
1^st^ IMAC	407	44	211	24	124	19
2^nd^ IMAC	162	18	76	9	40	6
